# Surface Tension of Cu-Ti Alloys and Wettability in a Liquid Alloy–Refractory Material-Gaseous Phase System

**DOI:** 10.3390/ma17194786

**Published:** 2024-09-29

**Authors:** Katarzyna Nowinska, Grzegorz Siwiec, Tomasz Matula, Alphonce Wikedzi, Beata Oleksiak, Jaroslaw Piatkowski, Tomasz Merder, Mariola Saternus

**Affiliations:** 1 Department of Electrical Engineering and Automation in Industry, Faculty of Mining, Safety Engineering and Industrial Automation, Silesian University of Technology, ul. Akademicka 2, 41-100 Gliwice, Poland; katarzyna.nowinska@polsl.pl; 2 Department of Metallurgy and Recycling, Faculty of Materials Science, Silesian University of Technology, Krasinskiego 8, 40-019 Katowice, Poland; grzegorz.siwiec@polsl.pl (G.S.); tomasz.merder@polsl.pl (T.M.); mariola.saternus@polsl.pl (M.S.); 3 Department of Mining and Mineral Processing Engineering, School of Mines and Geosciences, University of Dar es Salaam, Dar es Salaam P.O. Box 35131, Tanzania; awikedzi@udsm.ac.tz; 4 Department of Production Engineering, Faculty of Materials Science, Silesian University of Technology, Krasinskiego 8, 40-019 Katowice, Poland; beata.oleksiak@polsl.pl; 5 Department of Material Technologies, Faculty of Materials Science, Silesian University of Technology, Krasinskiego 8, 40-019 Katowice, Poland; jaroslaw.piatkowski@polsl.pl

**Keywords:** surface tension, contact angle, wettability, sessile drop method, high-temperature microscope, liquid Cu-Ti alloys

## Abstract

The study involved measurements of surface tension of liquid binary copper-titanium alloys with respect to their chemical composition and temperature as well as investigations of the liquid alloy–refractory material-gaseous phase system wettability using usual refractory materials, i.e., graphite, aluminum oxide and magnesium oxide. The experiments were performed with the use of the sessile drop method and a high-temperature microscope coupled with a camera and a computer. The aim of this study was to determine the influence of titanium content in the Cu-Ti alloy on the surface tension and contact angle at the interface between the liquid alloy and the refractory material. The influence of temperature on these parameters was also examined. The tests were carried out for copper-titanium alloys with a maximum content of 1.5% wt. Ti, in the temperature range of 1373 to 1573 K. The test results indicate that as the titanium content in the alloy increases, its surface tension increases slightly. However, an increase in temperature causes a decrease in the surface tension of the alloys. In the case of an alloy containing 1.5% wt. Ti, surface tension at a temperature of 1373 K reaches 1351 mN∙m^−1^, and at a temperature of 1573 K, it decreases to 1315 mN∙m^−1^. As the temperature and titanium content in the alloy increase, a decrease in the contact angle is observed. The highest values of contact angles were recorded in the case of contact of the liquid alloy with graphite. For an alloy containing 0.1% wt. Ti at a temperature of 1373 K, the contact angle reaches 132°, while at a temperature of 1573 K, it decreases to 128°. For an alloy containing 1.5% wt. Ti, the values of contact angles are 100° and 96°, respectively. However, the contact angles have the lowest values for magnesium oxide. In the case of a temperature of 1573 K and an alloy containing 1.5% wt. Ti, the contact angle reaches 49°. Such a significant impact of titanium content on the contact angles may be due to its high affinity for oxygen (contact with a substrate made of Al_2_O_3_ and MgO and its reactivity with carbon (contact with graphite).

## 1. Introduction

The surface tension of liquid metals and alloys and the wettability at the contact surface of the liquid phase and the solid phase (the size of the contact angle) have a significant impact on the phenomena occurring on interfacial surfaces during metallurgical processes for the production and refining of metals. Surface tension and wettability also play an important role, among others, during the processes of casting, welding, soldering, applying metallic coatings and obtaining composite materials. Those properties also play an important role in the phenomena related to the corrosion of refractory materials used in pyrometallurgical processes.

The knowledge of the surface tension of liquid metals and alloys, as well as the contact angles at the interface between the liquid and solid phases, in addition to the knowledge of, among others, the density or viscosity of the liquids, is now gaining new importance, especially in modeling metallurgical and foundry processes as well as modeling material properties [1,2,3].

Despite the continuous development of research methods in the case of high-temperature measurements of the surface tension of liquid metals and alloys as well as measurements of contact angles, a number of difficulties are still encountered and, among others, the appropriate selection of materials for making certain parts of the apparatus or substrate intended for placing samples and ensuring appropriate protective atmospheres and tightness. It is necessary to eliminate all chemical reactions among the components of the atmosphere, the tested liquid and the materials from which the parts of the measuring equipment are made. This leads to disturbances in the measurement of surface tension and contact angles.

This study involved measurements of surface tension of liquid binary copper-titanium alloys with respect to their chemical composition and temperature as well as investigations into the liquid alloy–refractory material-gaseous phase system wettability using usual refractory materials, i.e., graphite, aluminum oxide and magnesium oxide. The tests were carried out for copper-titanium alloys with a maximum content of 1.5% wt. Ti, in the temperature range of 1373 to 1573 K (most commonly used in industrial processes of manufacturing, refining and casting copper alloys). The research results obtained in this paper can be helpful, among other things, in the selection of refractory materials in the mentioned processes. It should be mentioned that high values of surface tension and contact angles are advantageous in pyrometallurgical manufacturing and refining processes because they reduce the possibility of corrosion of refractory materials and, thus, the possibility of contamination of the liquid metal with their components. On the other hand, for better shape mapping in casting processes, lower surface tension values and good wettability are advantageous.

The properties of Cu-Ti alloys have been of interest to researchers for years due to their potential use as an alternative to beryllium bronzes, which are characterized by high abrasion and corrosion resistance and are non-sparking materials [4,5,6,7,8,9,10]. Few publications on the surface tension of liquid copper-titanium alloys indicate that as the titanium content in the alloys increases, their surface tension increases [11,12,13]. However, the published data were based on alloys with a higher titanium content than in the presented work.

## 2. Materials and Methods

Pure copper (vacuum refined with a 99.9999% purity) and copper-titanium alloys with a content of 0.1, 0.25, 0.5, 0.75, 1, 1.25, 1.5% wt. Ti was used in the surface tension and wettability tests carried out in this work. Titanium, with a purity of 99.995%, was used to produce the alloys. Cylindrical samples with a diameter and height of approximately 4 mm were prepared from the above-mentioned alloys and pure copper. Graphite substrate was used for surface tension measurements. In wettability tests, in addition to graphite, substrates made of Al_2_O_3_ and MgO were also used.

The tests were carried out at temperatures of 1373, 1423, 1473, 1523 and 1573 K, and Argon, with a purity of 99.9999%, was used as a protective atmosphere during the tests.

**Figure 1:**
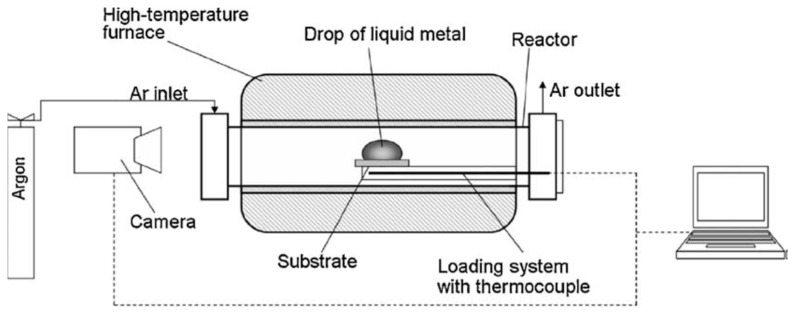
Schematic view of the apparatus.

The experiments were performed with the use of the sessile drop method. A high-temperature microscope coupled with a camera and a computer equipped with a program enabled the control of the device’s operating parameters, image recording and analysis. During the tests, the image of the sample was observed on the monitor and recorded on the computer’s hard drive, which allowed subsequent measurements of the appropriate geometric parameters of the liquid metal or alloy drop and, thus, the determination of the liquid drop volume (which, with the knowledge of the mass of the sample, allowed the determination of its density), surface tension and contact angle. A schematic of the measurement apparatus is shown in Figure 1.

**Figure 2:**
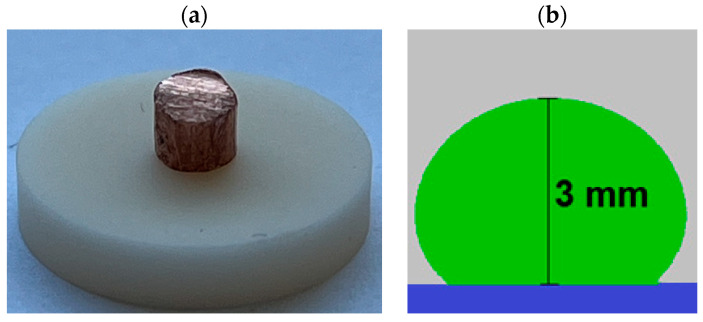
Sample shapes for surface tension and wettability testing: (**a**) example input specimen, (**b**) droplet shape of 0.25% wt. copper-titanium alloy. Ti at 1373 K on a graphite pad.

The sample to be tested, after being cleaned with ethyl alcohol and dried, was placed on a pad prepared in the same way. The pad, together with the sample, was then placed on the table of the loading unit, with the help of which it was introduced into the working chamber of the high-temperature microscope. After closing the measuring system, the reaction space of the furnace was purged with argon (flow rate of about 2.5∙10^−2^ m^3^/h) for about 2 h. Then, the sample was heated to the appropriate temperature. At the same time, the flow rate of argon was reduced to about 0.3∙10^−2^ m^3^/h. An example shape of the initial sample and a drop of liquid copper-titanium alloy recorded during the tests is shown in Figure 2.

To determine the surface tension of liquid copper and liquid Cu-Ti alloys, a computational procedure was used, which involved the estimation of the parameters of the differential equations describing the shape of a lying liquid drop using the least sum of squares method, based on the measured coordinates of points lying along the curve forming the outer outline of the drop’s cross-section. A detailed description of the research equipment and calculation procedure can be found in previous publications [3,14]. For each type of material for surface tension and wettability tests, measurements were repeated six times with identical experimental conditions.

## 3. Results and Discussion

The average results of measurements of the surface tension of liquid copper and liquid copper-titanium alloys are presented in Table 1. The results of measurements of the contact angle in the system: liquid alloy–refractory material-gas phase are presented in Table 2.

**Figure 3:**
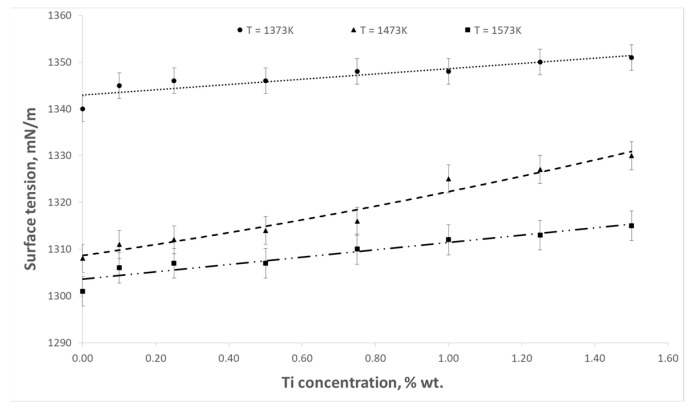
Surface tension isotherms of Cu-Ti alloys.

**Figure 4:**
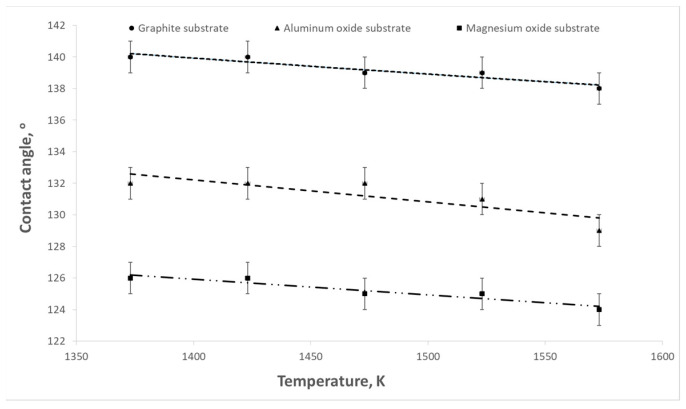
Changes in contact angles as a function of temperature for pure copper.

**Figure 5:**
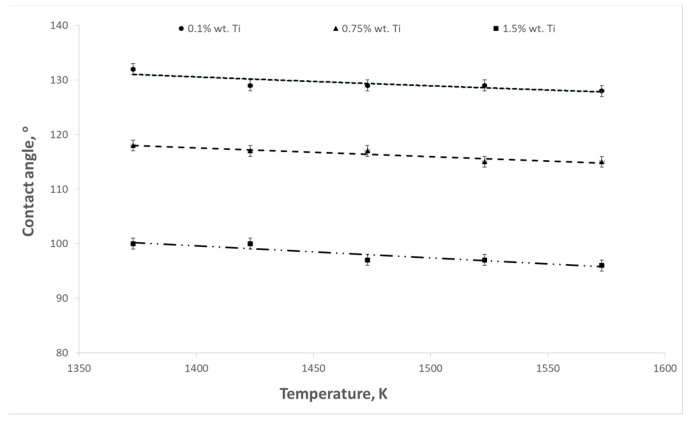
Changes in contact angles as a function of temperature for Cu-Ti alloys (graphite substrate).

**Figure 6:**
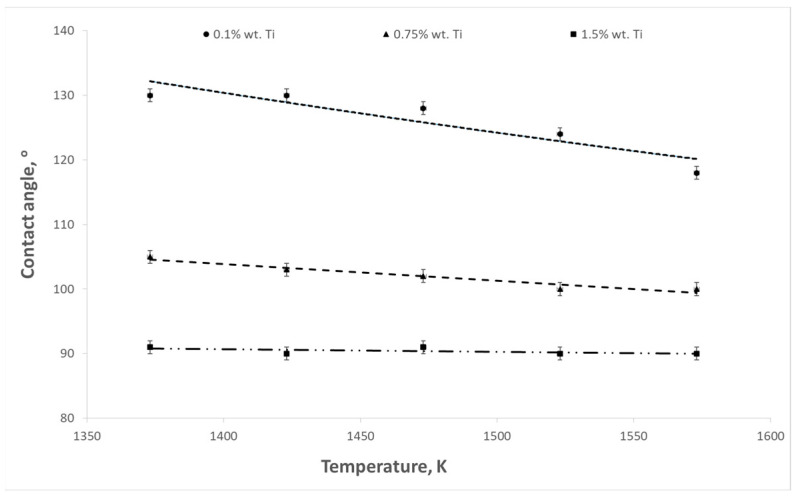
Changes in contact angles as a function of temperature for Cu-Ti alloys (Al_2_O_3_ substrate).

**Figure 7:**
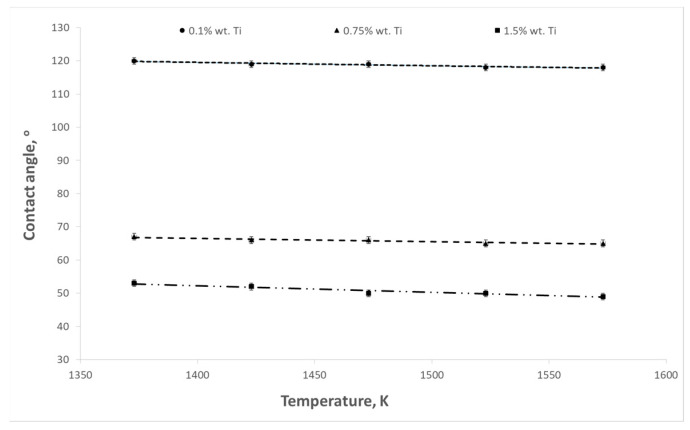
Changes in contact angles as a function of temperature for Cu-Ti alloys (MgO substrate).

**Figure 8:**
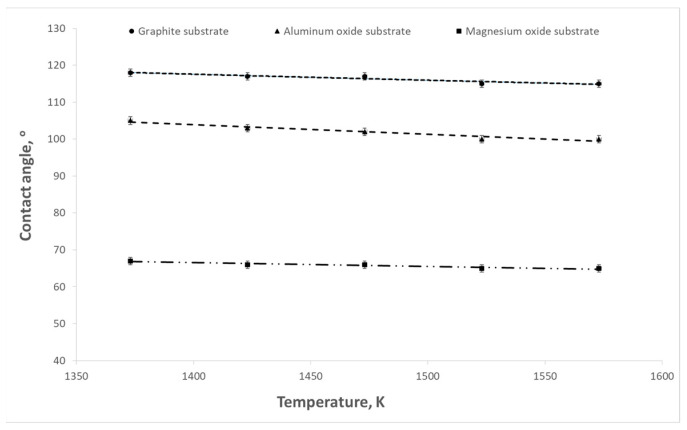
Changes in contact angles as a function of temperature for the Cu-Ti alloy (0.75% wt. titanium) depending on the type of substrate.

The aim of the presented study was to determine the influence of titanium content in the Cu-Ti alloy on the surface tension and contact angle at the interface between the liquid alloy and the refractory material. The influence of temperature on these parameters was also examined. Example results of surface tension and wettability tests are shown in Figure 3, Figure 4, Figure 5, Figure 6, Figure 7 and Figure 8.

The results obtained showed that in the range of titanium content analyzed in the paper, its addition to copper causes a slight increase in surface tension. For example, in the case of pure copper, the surface tension at 1373 K was 1340 mN∙m^−1^ and reached a value of 1351 mN∙m^−1^ for an alloy with 1.5% wt. Ti. On the other hand, at 1573 K, these values were 1301 mN∙m^−1^ and 1315 mN∙m^−1^, respectively. The increase in the surface tension of the alloy, along with an increase in Ti content, is presumably due to the higher surface tension of liquid titanium relative to copper, which reaches 1640 mN∙m^−1^ near the melting point [15]. The effect of an increase in surface tension with an increasing titanium content has also been observed in other publications on the surface tension of Cu-Ti liquid alloys [11,12,13]. This study further showed that for both pure copper and Cu-Ti alloys, an increase in temperature caused, in all cases, a decrease in surface tension. In the range of titanium content analyzed, increasing the temperature from 1373 K to 1573 K resulted in a decrease in the surface tension of 36–39 mN∙m^−1^.

As the temperature and titanium content in the alloy increased, a decrease in the contact angle was observed. The highest values of contact angles were recorded in the case of the contact of the liquid alloy with graphite (Figure 5). For an alloy containing 0.1% wt. Ti at a temperature of 1373 K, the contact angle reached a value of 132°, while at a temperature of 1573 K, it decreased to 128°. For an alloy with a content of 1.5% wt. Ti, these values were 100 and 96°, respectively. However, the contact angles have the lowest values for magnesium oxide (Figure 7). In the case of a temperature of 1573 K and an alloy containing 1.5% wt. Ti, the contact angle reaches 49°.

Pure titanium wets oxide surfaces very well [16]. As a component of alloys, it improves wettability [16,17]. Such a significant effect of titanium on the size of the wetting angles may be due to its high chemical affinity in the liquid state for oxygen (contact with the substrate made of Al_2_O_3_ and MgO). At the interfacial surface of the liquid alloy/solid metal oxide, a chemical reaction can occur between the titanium component of the alloy and the oxygen contained in the metal oxide of the substrate [16]. The authors of the paper [17], who studied the wettability of alumina with Cu-Pd-Ti alloys, found the appearance of a Ti_2_O_3_ layer at the copper/Al_2_O_3_ alloy interfacial interface. Oxygen, thus entering the liquid copper alloy, which is generally considered to be a surfactant [18], may have reduced the wetting angles. A similar mechanism may occur when liquid titanium-containing alloys come into contact with a magnesium oxide substrate. This is also confirmed by studies [19,20] on the melting process of titanium and titanium alloys in a vacuum induction furnace in crucibles made of alumina and magnesium oxide, among other materials. As a result of conducting these studies, crucibles were found to degrade during melting and the oxygen content of the titanium and its alloys increased after the processes.

In turn, the wettability improvement associated with the presence of titanium in alloys of a substrate made of graphite can be justified by the high reactivity of titanium in the liquid state with carbon. In the works [21,22], whose authors studied the wettability of graphite substrates with copper alloys with different titanium contents and found a decrease in the values of the contact angles with an increase in the titanium content in the alloys, they demonstrated the formation on the interfacial surface of liquid alloy-graphite of a layer made of titanium carbide. This is formed by a chemical reaction between the titanium in the liquid alloys and the carbon from the substrate. An analogous process was observed in studies of the melting process of titanium and titanium alloys in a vacuum induction furnace in crucibles made of graphite [23,24]. The experiments showed the degradation of graphite crucibles during melting and an increase in the carbon content of titanium and its alloys after the processes.

## 4. Conclusions

The measurements of surface tension of liquid copper and liquid copper-titanium alloys were investigated. The results indicated that the titanium contained in copper for the analyzed concentration range (up to 1.5% by mass) only slightly affected the surface tension value (minimum increase occurs). Temperature reduced the surface tension of Cu-Ti alloys, similar to pure copper.

Titanium content in the alloy was an element that had a significant impact on the wettability of the refractory materials used in the tests. The second factor influencing the size of the contact angles at the interface between the liquid alloy and the refractory material was temperature. Its increase always caused a decrease in the values of the measured contact angles.

The introduction of titanium into the alloy in the amount of 1.5% wt. reduced the contact angle on the Al_2_O_3_ surface to 90–91°. In the case of contact with MgO, the same titanium content caused a reduction in contact angles to even lower values, i.e., 53° at a temperature of 1373 K and 49° at a temperature of 1573 K. The contact angles in the system liquid copper alloy with titanium-graphite-gas phase also decreased with an increase in the Ti content in the alloy, reaching values in the range of 96 to 100° with the content of this element at the level of 1.5% by mass.

Such a significant impact of titanium content on the contact angles may be due to its high affinity with oxygen (contact with a substrate made of Al_2_O_3_ and MgO) and its reactivity with carbon (contact with graphite).

## Figures and Tables

**Table 1:** Results of surface tension measurements of liquid Cu-Ti alloys.

Ti Concentration, % wt.	Temperature, K	Density, kg∙m^−3^	Average Surface Tension, mN∙m^−1^
0	1373	8002	1340
	1423	7972	1325
	1473	7932	1308
	1523	7893	1305
	1573	7855	1301
0.10	1373	8000	1345
	1423	7970	1328
	1473	7930	1311
	1523	7891	1307
	1573	7850	1306
0.25	1373	7990	1346
	1423	7970	1329
	1473	7920	1312
	1523	7891	1308
	1573	7850	1307
0.50	1373	7890	1346
	1423	7900	1329
	1473	7880	1314
	1523	7880	1309
	1573	7845	1307
0.75	1373	7875	1348
	1423	7870	1331
	1473	7866	1316
	1523	7850	1311
	1573	7841	1310
1.00	1373	7860	1348
	1423	7858	1333
	1473	7850	1325
	1523	7841	1314
	1573	7837	1312
1.25	1373	7844	1350
	1423	7840	1339
	1473	7837	1327
	1523	7835	1320
	1573	7831	1313
1.50	1373	7840	1351
	1423	7829	1340
	1473	7824	1330
	1523	7819	1323
	1573	7811	1315

**Table 2:** Contact angle measurements in the system liquid Cu-Ti alloy–refractory material-gas phase.

Ti Con., % wt.	Temp., K	Graphite, °	Aluminum Oxide, °	Magnesium Oxide, °
0	1373	140	132	126
	1423	140	132	126
	1473	139	132	125
	1523	139	131	125
	1573	138	129	124
0.1	1373	132	130	120
	1423	129	130	119
	1473	129	128	119
	1523	129	124	118
	1573	128	118	118
0.25	1373	128	117	108
	1423	128	117	106
	1473	127	115	104
	1523	126	113	104
	1573	126	118	101
0.5	1373	120	109	90
	1423	120	109	89
	1473	118	108	88
	1523	118	107	87
	1573	117	107	86
0.75	1373	118	105	67
	1423	117	103	66
	1473	117	102	66
	1523	115	100	65
	1573	115	100	65
1.00	1373	110	96	63
	1423	110	95	82
	1473	108	95	60
	1523	108	94	60
	1573	106	93	59
1.25	1373	101	93	58
	1423	101	93	58
	1473	99	92	57
	1523	99	92	57
	1573	98	92	55
1.50	1373	100	91	53
	1423	100	90	52
	1473	97	91	50
	1523	97	90	50
	1573	96	90	49

## Data Availability

Data are contained within the article.
